# Nutrition support practices in critically ill head injured patients: a global perspective

**DOI:** 10.1186/2197-425X-3-S1-A984

**Published:** 2015-10-01

**Authors:** L Costello, M Chapman, A Deane, K Lange, D Heyland

**Affiliations:** Discipline of Acute Care Medicine, University of Adelaide, Adelaide, Australia; Intensive Care Unit, Royal Adelaide Hospital, Adelaide, Australia; Discipline of Medicine, University of Adelaide, Adelaide, Australia; Clinical Evaluation Research Unit, Kingston General Hospital, Kingston, Canada

## Introduction

Head injury induces a hypermetabolic state with energy expenditure up to 200% usual values, yet there is a lack of epidemiological data describing the delivery of nutritional therapy to critically ill head-injured patients. Based on reports from other diagnostic groups it is likely that feeding of head-injured patients varies greatly between institutions and countries. Furthermore, there are limited data describing associations between nutritional therapy in critically ill head-injured patients and clinical outcomes.

## Objectives

(1) To describe current global nutrition practices after head injury in the first 12 days of ICU admission;

(2) to determine factors that influence nutrition delivery; and

(3) to explore the relationships between energy and protein intake and clinical outcomes in this cohort.

## Methods

Retrospective analysis of observational data collected prospectively as part of the International Nutrition Survey from 2007-2013 was conducted for patients with a diagnosis of head trauma. Data are mean (SD), median [IQR], or percentages. Nutritional deficit was calculated as the mean daily absolute difference between intake and prescribed requirements. Pearson correlation was used to assess linear relationships between continuous variables. Associations with mortality and nutrition intake were determined via logistic regression and linear mixed effects models, respectively, adjusted for age, sex, region, APACHE II, BMI category, admission category, evaluable nutrition days and clustering of patients within ICUs. Cox regression was used to analyse time to discharge alive from ICU/hospital and length of mechanical ventilation, with inpatient death defined as a competing event.

## Results

Data for 1045 of 17689 patients from 341 sites were analysed. Patients had a mean age of 44.5(19.7), 78% were male, and median length of stay in ICU and hospital were 13.1[7.9-21.6] and 29.7[17.9-57.1]days. Most patients (94%) were enterally fed with a mean time to initiate nutritional therapy of 35.5(32.7)hours; longer time to initiation was associated with increased energy (r = 0.32) and protein (r = 0.27) deficit (both p < 0.001). Mean prescribed requirements were 25.9(4.9)kcal/kg/day and 1.29(0.3)grams protein/kg/day. 58% of energy and 53% of protein requirements were provided to patients. Patients from an ICU that utilised a feeding protocol had greater energy and protein intakes than those without (p < 0.001, 0.002 respectively) and were more likely to survive (OR0.65; 95%CI 0.42-0.99; p = 0.043). A greater energy and protein deficit was associated with longer time until discharge alive from ICU and hospital, and time receiving mechanical ventilation (all p < 0.001). There was no relationship between energy and protein intake and mortality (OR1.17, 95%CI 0.85,1.63, p = 0.335; OR1.02, 95%CI 0.96,1.08, p = 0.529, respectively).

## Conclusions

Critically ill head-injured patients develop nutritional deficits, but these may not be associated with increased mortality.

